# Gun violence and the voices of youth on community safety in the time of COVID-19 in East Harlem, NY: a youth participatory action research cross-sectional study

**DOI:** 10.1186/s40621-023-00440-x

**Published:** 2023-07-12

**Authors:** Jacqueline G. Wallace, Rachel Chernet, Margaret K. Formica, Olusola Adeonigbagbe, Roseanne L. Flores, Robert Marchesani, Danielle Goldberg, Pamela Wridt, Danielle Laraque-Arena

**Affiliations:** 1 Icahn School of Medicine at Mount Sinai, New York, NY USA; 2 SUNY Upstate Medical University, Syracuse, NY USA; 3 Hunter College CUNY, New York, NY USA; 4 Counseling In Schools Based at the Heritage School, New York, NY USA; 5 UNICEF USA, New York, NY USA; 6 Conscious Data Inc., Brooklyn, NY USA; 7 New York Academy of Medicine, 1216 Fifth Avenue, New York, NY 10029 USA; 8 Mailman School of Public Health and Vagelos College of Physicians and Surgeons, Columbia University, New York, USA

**Keywords:** Child rights, Gun violence, Youth, Community safety, YPAR, Participatory action research, CFCI, Youth voice

## Abstract

**Background:**

The USA has failed to codify the protection of children from gun violence (GV) as a human right. This study employs a youth participatory action research methodology, within the framework of the United Nations Convention on the Rights of the Child (UNCRC), to investigate the relationships between GV exposure, self-identified gender and perceptions of children’s rights and safety.

**Methods:**

An anonymous survey based on UNICEF USA’s Child Friendly Cities Initiative interactive survey tool targeting adolescents was modified by East Harlem, New York high school student co-researchers in collaboration with near-peer graduate students. The 61-question survey was administered at an East Harlem high school. Analysis consisted of univariate, bivariate and logistic regression using SPSS®.

**Results:**

A total of 153 students completed the survey: 48.4% self-identified as male and 45.8% as female. Thirty-five percent reported witnessing GV. Most (79.1%) were aware of child rights regardless of gender or GV exposure but there were differences in perceptions of safety. Fifteen percent of females reported never feeling safe at school compared to 3% of males (*p* = 0.01). Females were 2.2 times as likely as males to report transportation waiting areas as never safe (*p* = 0.008). Almost a third of females reported never feeling safe from sexual harassment in public, compared to 10% of males (*p* = 0.004). In multivariable logistic regression adjusted for gender, race/ethnicity and grade level, students who witnessed GV were 4.6 times more likely to report never feeling safe from violence (95% CI 1.7–12.4). Thirty percent of students who witnessed GV reported not attending school because of safety concerns. Students who witnessed GV had 2.2 times the odds of carrying a weapon to school (95% CI 1.1–4.5). These patterns continued for other perceptions of safety.

**Conclusions:**

The students in this study affirmed their rights to participate and express their views on matters that may affect them, as articulated in the UNCRC. The study revealed differences in perceptions of safety by self-identified gender and identified gun violence as a major contributor of youth’s perception of lack of safety. The study evinces the efficacy of employing YPAR methodology to identify and answer youth concerns of community safety and prioritize honoring child rights.

## Background

The United Nations (UN) Convention on the Rights of the Child (CRC) is a legally binding international human rights treaty that acknowledges that children have the same general human rights as adults as well as specific rights unique to the needs of children (Kilbourne 1998). The CRC is the most widely ratified human rights treaty in the world, but the USA has not ratified it. Article 6 of the CRC recognizes that every child has the inherent right to live and Article 19 states “parties shall take all appropriate legislative, administrative, social and educational measures to protect the child from all forms of physical or mental violence” (United Nations 1989). The USA has failed to institute policies that codify and recognize that the protection of children from direct and indirect harm of gun violence (GV) is a human right (United Nations 1989). In 2020, firearm injuries surpassed motor vehicle crashes as the leading cause of death for children ages 1–19 in the USA, reflecting a dramatic increase in GV during the COVID-19 pandemic (Goldstick et al. 2022). The relative increase in the rate of firearm-related deaths among children from 2019 to 2020 was nearly 30%, which is more than twice as high as the relative increase in the general population (Goldstick et al. 2022). The physical consequences of being a target of GV are obvious, but the repercussions to children who are exposed to GV in their communities are more insidious: there are significant biological, psychological and social consequences to children who witness GV (Jennissen et al. 2021; Wintemute et al. 2022; Smith et al. 2020; Pinheiro 2006).

Article 12 of the CRC emphasizes that children are capable of forming their own views and have the right to express those views freely in all matters affecting the child (United Nations 1989). Ongoing efforts to address issues related to GV too often fail to incorporate the perspectives of children impacted by the violence. Voting is an integral way that citizens in a democracy can address societal issues that affect them, but children are largely excluded from the voting process, so there is a risk that without intentional efforts to include them, the views of children on issues such as GV will go unheard. Adopting a Child Rights Framework, as outlined by the CRC, to guide behavior, actions, policies, and programs, is an important strategy that can ensure children and youth, without discrimination, have opportunities to share their lived experiences and be agents of social change (United Nations 1989). Youth Participatory Action Research (YPAR) is an innovative, equity-focused research methodology that aligns well with a Child Rights Framework. Participatory action research disrupts the traditional research model in which researchers observe, analyze, and propose solutions to issues of a population the researchers themselves may not be a part of; instead, this research methodology empowers the very population being studied to be a part of the research process. In YPAR, academic researchers collaborate with youth as co-researchers. Youth are provided the opportunity to identify and study problems that affect them and to share their knowledge as experts of their own lives to determine actions to solve these problems through youth-adult partnerships (Anselma et al. 2020; Anderson 2020; Anyon et al. 2018).

The purpose of this study was to employ the YPAR methodology within a Child Rights Framework to better understand youth perceptions of safety in East Harlem (EH), New York City and explore relationships to GV exposure. A secondary aim of the study was to examine the relationship of gender and perceptions of safety since exposure to violence can be gender specific and prior studies have reported differences in the effects of exposure to violence based on gender (Saadatmand et al. 2018). Contextually, it is important that this study took place in the second year of the COVID-19 pandemic when students had returned to in-person classes. We directly collaborated with EH youth at The Heritage School (THS) to investigate school and community factors that affect their well-being with the aim to identify points of intervention previously neglected, to elucidate systemic issues that impact EH among both youth and decision-makers, and to derive lessons that may translate to youth in other communities.

## Methods

### Participants

#### YPAR: youth participation in the research process

Three high school students from THS in EH were selected by the school for an internship to participate in the research process. The students contributed to survey design, survey implementation and presentation of results. Video-conferencing technology enabled youth participation and research collaboration. Students received a stipend of $500 for their participation, which consisted of eight one hour Zoom meetings over the course of 3 months with two graduate students from the research group, and one student presentation in December 2021 to Manhattan Community Board 11 in EH that advises elected officials on matters affecting the social welfare of EH. The high school students were invited to but unable to attend consistently the bi-weekly Zoom meetings with the larger advisory research group, so the graduate students shared input from the three high school students at the meetings. The advisory group, the Intergenerational Action Adolescent and Child Team (IAACT), founded by Laraque-Arena, is composed of researchers and representatives from the New York Academy of Medicine, THS, Counseling In Schools, UNICEF USA, Columbia University, the City University of New York, Icahn School of Medicine at Mount Sinai, and SUNY Upstate Medical University. The survey was finalized with input from the students in discussion with IAACT.

#### Survey population

The study population consisted of 154 students from THS in EH who attended advisory period. All students were eligible to participate and were offered a $5 incentive to participate which they received upon completion of the survey. We reported the demographics of the student population since we examined grade level, gender, and racial/ethnic perspectives regarding community safety that may differ by these characteristics.

### Survey development

This study was part of a 2-year study which collected data in March 2021 and April 2022. The preliminary Year 1 study focusing on the survey development and modifications is presented in this supplement and can be referenced for more details (Malla et al. 2023). In short, the survey for the present Year 2 study was adapted from the UNICEF USA Child Friendly Cities Initiative (CFCI) Community interactive survey tool targeting adolescents, adapted from child-friendly assessment tools developed by the Children’s Environmental Group (CERG, 2014) at the City University of New York (CUNY). UNICEF USA uses these developmentally appropriate tools within its Child Friendly Cities Initiative program to aid local governments to engage children and young people and their adult allies in child right-based conversations of their community conditions and the extent to which children’s rights are being realized, as laid out in the CRC. The 34 CFCI questions were within 5 domains. The domain “My participation” includes 10 questions about students’ involvement in their school and community. The domain “My Living Environment” includes six questions about students’ perceptions of where they live. The domain “My Community Services” includes 11 questions about students’ awareness of and access to resources in their community. The domain “My Play and Leisure” includes 10 questions about students’ perceptions of accessibility to play spaces and perceptions of safety in the play spaces and around their community. The domain “My Safety and Inclusion” includes 11 questions about student’s perception of safety in their community (Lee et al. 2022b). The initial survey development process is detailed by Wridt and resulted in an age and content valid tool which was also extensively field tested in numerous countries in Africa, the Middle East, Asia and the USA (Wridt 2015; Wridt et al. 2015). This initial survey (considered required elements) was modified by this research group in collaboration with five high school students from THS during Year 1, 2020–2021. Following detailed discussions with Wridt, UNICEF USA’s Senior Consultant for CFCI and originator of the broader survey, the adolescent group added 14 questions derived from the broader UNICEF field study. This modified survey (inclusive of the 34 original questions) was pilot tested in Year 1. In addition, a number of validated questions were added from the CDC’s Youth Risk Behavior Surveillance Survey (YRBSS), which is an anonymous biennial voluntary survey of high school students (Centers for Disease Control and Prevention 2019). The validated questions from the YRBSS related to safety, violence, weapon use and mental health during COVID-19. In Year 2, 2021–2022, the modified survey was further revised in collaboration with the three THS students. The goal of the revision process was to gather student input on whether the survey questions were relevant to their lives and phrased using language that was understandable to the students within the constructs laid out by UNICEF USA’s CFCI interactive survey process. The students advocated to remove the survey response option of “I don’t know” and add the response options “I prefer not to answer” and “I don’t understand the question.” The survey tool required that every question be answered, so students were given the choice to select “I prefer not to answer” if they wished to skip a question. (It should be noted that in the global experience with the CFCI interactive survey process, the quantitative survey administration was followed by robust focus group discussions that served to further illuminate youth concerns.)

The final survey consisted of 61 questions: 48 questions modified from the CFCI interactive survey tool, six questions from the YRBSS, five demographic questions, one question about experience with GV, and one open ended question. The survey was uploaded to QuestionPro®, a free online survey software development tool, so that the survey could be accessed and completed anonymously via a link from any device with internet access. The survey is accessible via this website: https://questionpro.com/t/AVBKdZvpKb.

### Survey implementation

The survey was administered in person at THS during 14 student advisory sessions over the course of 4 days, April 5–8, 2022. Students were offered the option to complete the survey during the advisory period on their own or the school’s electronic devices. A short discussion session followed. The survey link remained open for a period of 7 days to allow any student who did not complete the survey during the 45 min class time to participate.

### Analytic approach

For the purpose of this study, we focused the analysis on a selection of the survey questions that related to child rights and safety.

SPSS® was used for analysis. Univariate frequencies and proportions were obtained for the responses to each question: Mostly True, Sometimes True, Never True, I don’t understand the question, and I prefer not to answer.

Grade level was determined based on the time the survey was completed since the surveys were administered in grade-specific advisory sessions. One student completed the survey outside of class time, so the grade level was not determined for that student.

To describe the socioeconomic status of the students at THS, we used Child Opportunity Levels (COLs) from the Child Opportunity Index, which measures and maps the quality of resources and conditions that matter for children. The levels include very low, low, moderate, high, and very high. COLs were determined from a database from the diversitydatakids.org titled Child Opportunity Index 2.0 ZIP code index and population data aggregated to 2020 USPS ZIP codes (Diversitydatakids.org 2021).

To evaluate whether responses differed by whether the student had witnessed GV, bivariate analyses were performed examining the distribution of answers in response to the question “Have you ever personally witnessed gun violence?” A similar analysis was completed to determine if responses differed by self-identified females compared to self-identified males for select questions related to child rights and perceptions of safety. The responses “I don’t understand the question” and “I prefer not to answer” were excluded. Because of the limited sample size of the study, the response categories were collapsed into either never true vs. sometimes/mostly true or mostly true vs never/sometimes true. To limit bias, the decision of how to collapse the response categories was determined prior to analysis. Statistical comparisons across groups were made using Chi-square statistics. Two-tailed statistical significance was assessed at *p* < 0.05.

Multivariable logistic regression models controlling for gender, self-identified race/ethnicity, and grade level were used to evaluate the association between exposure to GV and the questions in the survey that related to child rights and safety from the survey sections “My Participation,” “My Play and Leisure” and “My Safety and Inclusion.” For this analysis due to the low number of students who identified as Asian/Asian American, White/Caucasian, and Native American/Alaska Native, these groups were combined together. For gender, the categories non-binary, transgender and prefer to self-describe were combined. Odds ratios with corresponding 95% confidence intervals were obtained to quantify association between exposure to gun violence and perceptions of safety.

## Results

During the week, the survey was administered, the baseline enrollment at THS was 292 students with a daily mean of 244 students in attendance at the school during the survey period, and a total of 154 students attended advisory sessions. All students in advisory sessions elected to participate; one student was unable to complete the survey due to internet connectivity issues yielding 153 completed surveys for analysis (response rate of 62.7%).

Characteristics of the survey respondents are shown in Table 1. Forty-eight percent of students self-identified as male, 45.8% female, with the remaining 6.0% choosing either non-binary, transgender or preferred not to say. Forty-two and a half percent of respondents stated they were Latinx, 30.1% Black/African American, 19.6% other, 5.6% Asian/Asian American, 2.0% White/Caucasian, and 1.3% Native American/Alaska Native. A total of 33 freshmen, 37 sophomores, 28 juniors, 54 seniors, and one student whose grade level was not identified completed the survey. The majority of students reported living either in the boroughs of Manhattan (58.2%) or the Bronx (28.1%). The majority lived in zip codes that had an overall COL level of very low (78.8%) or low (5.2%) with only one student who lived in a zip code rated as very high. Of note, THS is in zip code 10029, which had an overall COL level of very low.

**Table 1:** Characteristics of survey respondents

	*N* (%)
Gender (*N* = 153)	
Male	74 (48.4%)
Female	70 (45.8%)
Prefer not to answer	5 (3.3%)
Non-binary	3 (2.0%)
Transgender	1 (0.7%)
Prefer to self-describe	0 (0.0%)
Race/ethnicity (*N* = 153)	
Latinx	65 (42.5%)
Black/African American	46 (30.1%)
Other	30 (19.6%)
Asian/Asian American	7 (4.6%)
White/Caucasian	3 (2.0%)
Native American/Alaska Native	2 (1.3%)
Year in school (*N* = 153)	
Senior	54 (35.3%)
Sophomore	37 (24.2%)
Freshman	33 (21.6%)
Junior	28 (18.3%)
Unknown	1 (0.7%)
Zip codes (*N* = 153)	
Manhattan (10001–10282)	89 (58.2%)
Bronx (10451–10475)	43 (28.1%)
Queens (11004–11109, 11351–11697)	5 (3.3%)
Brooklyn (11201–11256)	4 (2.6%)
New Rochelle (10805)	1 (0.7%)
Not reported	11 (7.2%)
Overall child opportunity level (*N* = 153)	
Very high	1 (0.7%)
High	7 (4.6%)
Moderate	3 (2.0%)
Low	8 (5.2%)
Very low	119 (77.8)
Missing zip code or COL not reported	15 (9.8%)

### Overall survey results

The overall frequencies of responses to the select survey questions related to child rights and safety are summarized below according to the five survey domains of the CFCI interactive survey (student participation, student living environments, community services, play and leisure, and safety and inclusion) as well as the YRBSS questions. Select survey results are displayed in Table 2. The CFCI questions included an option to select “I prefer not to answer” for those students who wished to skip the question. The total percentage of questions that were skipped was 3%.

**Table 2:** East Harlem High School students' responses to selected survey questions related to child rights and safety (*N* = 153), April 2022

	Mostly true	Sometimes true	Never true	I don't understand the question	I prefer not to answer
*My participation*					
I am aware that children have rights	121 (79.1%)	28 (18.3%)	2 (1.3%)	0 (0.0%)	2 (1.3%)
I have open opportunities to voice my ideas and concerns about decisions that affect me in New York City	39 (25.5%)	80 (52.3%)	28 (18.3%)	2 (1.3%)	4 (2.6%)
I have open opportunities to give my opinion about school decisions and my voice makes a difference	50 (32.7%)	65 (42.5%)	28 (18.3%)	1 (0.7%)	9 (5.9%)
I contribute to projects to change my community around the Heritage School in East Harlem	25 (16.3%)	67 (43.8%)	45 (29.4%)	2 (1.3%)	14 (9.2%)
I am involved in planning or decision-making for my community around the Heritage School in East Harlem	21 (13.7%)	55 (35.9%)	63 (41.2%)	2 (1.3%)	12 (7.8%)
I help lead actions to change the Heritage School, East Harlem, or New York City	26 (17.0%)	50 (32.7%)	67 (43.8%)	3 (2.0%)	7 (4.6%)
Adults act on and listen to my views or priorities for change in my school, East Harlem, or New York City	30 (19.6%)	75 (49.0%)	30 (19.6%)	8 (5.2%)	10 (6.5%)
I try to learn about political issues that affect young people in East Harlem	36 (23.4%)	82 (53.6%)	26 (17.0%)	6 (3.9%)	3 (2.0%)
*My living environments*					
I feel safe at home	140 (91.5%)	11 (7.2%)	1 (0.7%)	0 (0.0%)	1 (0.7%)
The air around me in my home is clean (free of toxins, dust, smoke, mold, etc.)	114 (74.5%)	28 (18.3%)	9 (5.9%)	1 (0.7%)	1 (0.7%)
The air around me in East Harlem is clean	23 (15.0%)	90 (58.8%)	32 (20.9%)	4 (2.6%)	4 (2.6%)
There are places in the Heritage School and in East Harlem that are clean, and pose little to no risks to my health	42 (27.5%)	83 (54.2%)	18 (11.8%)	6 (3.9%)	4 (2.6%)
*My community services*					
I am happy with the way my teachers interact with me and teach me	67 (43.8%)	70 (45.8%)	9 (5.9%)	2 (1.3%)	5 (3.3%)
I am content with how online school went last year	63 (41.2%)	59 (38.6%)	22 (14.4%)	3 (2.0%)	6 (3.9%)
I have access to the Internet at school or in East Harlem	87 (56.9%)	44 (28.8%)	16 (10.5%)	3 (2.0%)	3 (2.0%)
*My play and leisure*					
There are spaces for play and relax outside near my home that I use (e.g., parks, basketball courts, etc.)	105 (68.6%)	38 (24.8%)	8 (5.2%)	1 (0.7%)	1 (0.7%)
The play spaces in East Harlem are in good condition	55 (35.9%)	76 (49.7%)	18 (11.8%)	1 (0.7%)	3 (2.0%)
I have safe places for play, such as games and sports in East Harlem	75 (49.0%)	62 (40.5%)	11 (7.2%)	1 (0.7%)	4 (2.6%)
I feel safe using buses or other public vehicles	41 (26.8%)	85 (55.6%)	23 (15.0%)	1 (0.7%)	3 (2.0%)
I am safe from sexual harassment when in public spaces (e.g., hooting, cat calling, staring, etc.)	57 (37.3%)	58 (37.9%)	29 (19.0%)	2 (1.3%)	7 (4.6%)
It is safe for me to ride my bike in East Harlem	49 (32.0%)	67 (43.8%)	16 (10.5%)	7 (4.6%)	14 (9.2%)
I feel safe walking to school	81 (52.9%)	60 (39.2%)	8 (5.2%)	1 (0.7%)	3 (2.0%)
Public spaces are free from drug dealing and other illegal activities in East Harlem	28 (18.3%)	65 (42.5%)	44 (28.8%)	7 (4.6%)	9 (5.9%)
Local transportation waiting areas are safe (well-lit and clean)	33 (21.6%)	72 (47.1%)	39 (25.5%)	3 (2.0%)	6 (3.9%)
*My safety and inclusion*					
I feel safe from violence (protected from abuse, gangs, armed groups etc.) in the community around the Heritage School in East Harlem	50 (32.7%)	73 (47.7%)	22 (14.4%)	2 (1.3%)	6 (3.9%)
All genders are treated equally and given the same opportunities	57 (37.3%)	69 (45.1%)	21 (13.7%)	3 (2.0%)	3 (2.0%)
I feel safe from being bullied by other children at school	83 (54.2%)	51 (33.3%)	12 (7.8%)	1 (0.7%)	6 (3.9%)
I feel safe from being bullied by other children online	92 (60.1%)	42 (27.5%)	11 (7.2%)	2 (1.3%)	6 (3.9%)
I feel safe socially/physically/emotionally at school	68 (44.4%)	63 (41.2%)	14 (9.2%)	1 (0.7%)	7 (4.6%)
I have friends of different origins, backgrounds, genders, or abilities	118 (77.1%)	24 (15.7%)	5 (3.3%)	1 (0.7%)	5 (3.3%)
I trust police officers and feel they are allies in East Harlem	38 (24.8%)	77 (50.3%)	24 (15.7%)	2 (1.3%)	12 (7.8%)
I feel security guards in my school keep me safe	45 (29.4%)	70 (45.8%)	19 (12.4%)	4 (2.6%)	15 (9.8%)
There are adults at the Heritage School or in East Harlem who I can talk to freely about abuse or violence	59 (38.6%)	58 (37.9%)	23 (15.0%)	4 (2.6%)	9 (5.9%)

Experience	Yes	No			
I have personally witnessed gun violence	53 (34.6%)	100 (65.4%)			
*YRBSS*					
During the time you have attended The Heritage School, have you ever not attended school because you felt you would be unsafe at school?	29 (19.0%)	124 (81.0%)			
During the time you have attended The Heritage School, have you ever not attended school because you felt you would be unsafe going to or from school?	27 (17.6%)	126 (82.4%)			
Have you ever carried a weapon such as a gun, knife, or club on school property?	10 (6.5%)	143 (93.5%)			
Have you ever seen someone get physically attacked, beaten, stabbed, or shot in your neighborhood?	78 (51.0%)	75 (49.0%)			

YRBSS	Always	Most of the time	Sometimes	Rarely	Never
During the COVID-19 pandemic, how often was your mental health not good? (Poor mental health includes stress, anxiety, and depression.)	11 (7.2%)	37 (24.2%)	39 (25.5%)	34 (22.2%)	32 (20.9%)

YRBSS	Yes	No	My parents and adults in my home did not have jobs before COVID		
During the COVID-19 pandemic, did a parent or other adult in your home lose their job even for a short amount of time?	44 (28.8%)	100 (65.4%)	9 (5.9%)		

#### Student participation

Most students, 79.1%, were aware that children have rights. A similar percentage of students reported trying to learn about political issues that affect them most or some of the time (78.0%). Students shared that they have opportunities to voice their ideas and concerns about decisions that affect them in their community (77.8%) and school (75.2%), and that adults act on their views at least some of the time (68.6%). Most students (60.1%) felt they contributed to projects to change their community at least some of the time, few felt involved leading such projects. Students selected “never true” as the most frequent response when asked if they are involved in making decisions for their community (41.2%) or leading actions to change their community (43.8%).

#### Student living environments

Most students reported feeling safe at home (91.5%) and perceived the air in their homes to be free of toxins most of the time (74.5%). The perception of the cleanliness of the air in the community was not as favorable, with one in five students suggesting the air around EH is never clean. Students felt there are places in EH that are clean and pose little risk to their health most (27.5%) or some (54.2%) of the time.

#### Community services

Most students were happy with the ways their teachers interacted with them at least some of the time (89.6%). Only 14.4% of students were dissatisfied with how online school went during the first year of the COVID-19 pandemic. Internet access varied across the student body: 56.9% had access most of the time, 28.8% some of the time, and 10.5% reported never having access to the internet.

#### Play and leisure

Students used public spaces to play and relax outside their home most of the time (68.6%). Half of the students reported the play spaces were in good condition only sometimes, and more than 10% say they were never in good condition. When asked if the public spaces were free from drug dealing and illegal activities, the responses varied, with 18.3% saying mostly, 42.5% sometimes and 28.8% never. Students also varied in feeling safe from sexual harassment in public spaces: 37.3% mostly, 37.9% sometimes and 19.0% never.

Although most students felt comfortable using various forms of transportation to commute to school and around EH, there were students who never felt safe waiting in local transportation areas (25.5%) using buses (15.0%), riding their bike (10.5%), or walking to school (5.2%).

#### Safety and inclusion

When asked if students felt safe from violence in the community around their school, only 32.7% reported feeling safe most of the time. A slightly higher percentage of students (44.4%) felt safe socially/physically/emotionally at school most of the time. More than one third of the students felt safe from bullying only sometimes or never; either in-person (41.1%) or online (34.7%). The majority of students reported trusting security guards at school and police officers at least some of the time, but a not insignificant proportion of students felt school security guards do not keep them safe (12.4%) and reported they never trust police (15.7%). Of note, many students elected “prefer not to answer” for questions that related to adults providing protection of students including whether the students felt that school security guards kept the students safe (9.8%) and whether police officers can be trusted and are allies in EH (7.8%). Students varied in their perception of how often there was an adult they could talk freely to about violence and abuse: 38.6% report most of the time, 37.9% sometimes, and 15.0% never.

#### YRBSS questions

Thirty-five percent of students reported having personally witnessed gun violence. The mental health status of students varied; students reported poor mental health always (7.2%), most of the time (24.2%), sometimes (25.5%), rarely (22.2%) and never (20.9%). Almost one third (28.8%) of students reported that a parent or other adult in the home lost their job during the pandemic.

### Associations of gender with perceptions of child rights and safety

The results of the bivariate analysis to examine the association of gender and responses to select questions related to child rights and perceptions of safety are presented in Table 3.

**Table 3:** Association of gender and select survey questions related to child rights and safety

	Male	Female	*P* value
*My participation*			
I am aware that children have rights			0.9
Never	2%	2%	
Sometimes/mostly	98%	98%	
*My living environment*			
There are places in the Heritage School and in East Harlem that are clean, and pose little to no risks to my health			0.02
Never	6%	18%	
Sometimes/mostly	94%	81%	
The air around me in East Harlem is clean			0.02
Never	14%	31%	
Sometimes/mostly	86%	69%	
*My play and leisure*			
I have safe places for play, such as games and sports in East Harlem			0.02
Never	1%	11%	
Sometimes/mostly	99%	89%	
I feel safe using buses or other public vehicles			0.6
Never	13%	16%	
Sometimes/mostly	87%	84%	
I am safe from sexual harassment when in public spaces (e.g., hooting, cat calling, staring, etc.)			0.004
Never	10%	29%	
Sometimes/mostly	90%	71%	
It is safe for me to ride my bike in East Harlem			0.08
Never	7%	17%	
Sometimes/mostly	93%	83%	
I feel safe walking to school			0.08
Never	1%	7%	
Sometimes/mostly	99%	93%	
Public spaces are free from drug dealing and other illegal activities in East Harlem			0.05
Never	25%	41%	
Sometimes/mostly	75%	59%	
Local transportation waiting areas are safe (well-lit and clean)			0.008
Never	17%	37%	
Sometimes/mostly	83%	63%	
*My safety and inclusion*			
All genders are treated equally and given the same opportunities			0.02
Never	7%	21%	
Sometimes/mostly	93%	79%	
I feel safe socially/physically/emotionally at school			0.01
Never	3%	15%	
Sometimes/mostly	97%	85%	
There are adults at the Heritage School or in East Harlem who I can talk to freely about abuse or violence			0.03
Never/sometimes	50%	68%	
Mostly	50%	32%	
*YRBS*			
During the time you have attended The Heritage School, have you ever not attended school because you felt you would be unsafe at school?			0.03
Yes	11%	24%	
No	89%	76%	
During the time you have attended The Heritage School, have you ever not attended school because you felt you would be unsafe going to or from school?			0.2
Yes	12%	20%	
No	88%	80%	
Have you ever carried a weapon such as a gun, knife, or club on school property?			0.2
Yes	10%	4%	
No	90%	96%	
During the COVID-19 pandemic, how often was your mental health not good? (Poor mental health includes stress, anxiety, and depression.)			0.00002
Always	4%	10%	
Most of the time	11%	34%	
Sometimes	32%	20%	
Rarely	19%	29%	
Never	34%	7%	

#### Child rights and gender

The survey population included 70 students who self-identified as female and 74 who self-identified as male. Regardless of gender, students reported understanding that children have rights; however, females were three times as likely as males to report that genders are never treated equally and never given equal opportunities (21% females, 7% males, *p* = 0.02).

#### Perceptions of safety and gender

Females were significantly more likely to report never feeling safe in various situations, including at school. Fifteen percent of females reported never feeling safe socially, physically, or emotionally compared to 3% of males (*p* = 0.01). Significantly higher percentages of female students reported skipping school because they felt they would be unsafe at school (24% females, 11% males, *p* = 0.03). Although female and male students reported feeling safe when commuting to school at similar rates, females were 2.2 times as likely as males to report local transportation waiting areas as never being safe spaces (37% females, 17% males, *p* = 0.008). Almost a third of females reported never feeling safe from sexual harassment in public spaces, compared to only 10% of males who felt similarly (*p* 0.004).

Females were more likely than males to perceive their environment as unsafe. Forty-one percent of females reported feeling public spaces were never free from illegal activity, versus 25% of males (*p* = 0.05). Females were more likely than males to report that there are never safe places for play in EH (11% of females, 1% of males, *p* = 0.02). Almost 20% of females reported that there were never places at their school and around their community that are clean and pose little risk to health compared to only 6% of males who report the same (*p* = 0.02). Twice as many females as males believed the air around them in EH was never clean (31% of females, 14% of males, *p* = 0.02).

Forty-four percent of females reported that their mental health was poor most of the time or always, compared to only 15% of males who responded similarly (*p* = 0.00002). A lower percentage of females (32%) compared to males (50%) shared that they feel there is an adult they can speak freely to about abuse or violence most of the time (*p* = 0.03).

### Associations of witnessing gun violence with perceptions of child rights and safety

#### Demographics of students who witnessed gun violence

The demographic characteristics of students who witnessed GV were similar to the demographic characteristics of students who did not witness GV.

#### Child rights and gun violence

All students who witnessed GV, and 98% of those who didn’t were aware children have rights some or most of the time. There were no significant differences based on exposure to GV in the percentages of students who learned about political issues. Students who witnessed GV reported having opportunities to contribute, lead and make decisions in projects to change their communities at similar rates of students who had not witnessed GV. Students felt that they were able to voice their opinions and have adults act on them at least some of the time, regardless of exposure to GV. (Data not shown).

#### Perceptions of safety and gun violence

There were significant differences in student perceptions of safety in the community depending on whether the student witnessed GV. Results of the analyses which examine the association of witnessing GV and perceptions of safety are presented in Table 4.

**Table 4:** Association of gun violence and perceptions of safety

	Witness GV		Adjusted OR* (95%CI)
	Yes	No	
*My play and leisure*			
There are spaces for play and relax outside near my home that I use (e.g., parks, basketball courts, etc.)			1.2 (0.9–1.5)
Never/sometimes	24%	34%	
Mostly	76%	66%	
The play spaces in East Harlem are in good condition			0.9 (0.7–1.2)
Never/sometimes	65%	62%	
Mostly	35%	38%	
I have safe places for play, such as games and sports in East Harlem			0.8 (0.2–3.2)
Never	8%	7%	
Sometimes/mostly	92%	93%	
I feel safe using buses or other public vehicles			0.6 (0.2–1.7)
Never	12%	18%	
Sometimes/mostly	88%	82%	
I am safe from sexual harassment when in public spaces (e.g., hooting, cat calling, staring, etc.)			**3.7 (1.5–8.8)**
Never	32%	13%	
Sometimes/mostly	68%	87%	
It is safe for me to ride my bike in East Harlem			0.8 (0.3–2.6)
Never	11%	13%	
Sometimes/mostly	89%	87%	
I feel safe walking to school			2.2 (0.5–9.6)
Never	8%	4%	
Sometimes/mostly	92%	96%	
Public spaces are free from drug dealing and other illegal activities in East Harlem			1.6 (0.8–3.6)
Never	37%	29%	
Sometimes/mostly	63%	71%	
Local transportation waiting areas are safe (well-lit and clean)			**2.5 (1.1–5.6)**
Never	37%	22%	
Sometimes/mostly	63%	78%	
*My safety and inclusion*			
I feel safe from violence (protected from abuse, gangs, armed groups etc.) in the community around the Heritage School in East Harlem			**4.6 (1.7–12.4)**
Never	28%	8%	
Sometimes/mostly	72%	92%	
All genders are treated equally and given the same opportunities			2.2 (0.8–5.9)
Never	20%	11%	
Sometimes/mostly	80%	91%	
I feel safe from being bullied by other children at school			1.2 (0.4–4.2)
Never	0%	7%	
Sometimes/mostly	90%	93%	
I feel safe from being bullied by other children online			1.0 (0.3–3.7)
Never	8%	8%	
Sometimes/mostly	92%	92%	
I feel safe socially/physically/emotionally at school			1.7 (0.5–5.3)
Never	12%	8%	
Sometimes/mostly	88%	92%	
I trust police officers and feel they are allies in East Harlem			1.9 (0.7–4.7)
Never	24%	14%	
Sometimes/mostly	76%	86%	
I feel security guards in my school keep me safe			**0.6 (0.5–0.9)**
Never/sometimes	81%	58%	
Mostly	19%	42%	
There are adults at the Heritage School or in East Harlem who I can talk to freely about abuse or violence			**0.7 (0.5–0.9)**
Never/sometimes	70%	51%	
Mostly	30%	49%	
*YRBS*			
During the time you have attended The Heritage School, have you ever not attended school because you felt you would be unsafe at school?			**1.6 (1.1–2.6)**
Yes	30%	13%	
No	70%	87%	
During the time you have attended The Heritage School, have you ever not attended school because you felt you would be unsafe going to or from school?			1.4 (0.9–2.2)
Yes	24%	14%	
No	76%	86%	
Have you ever carried a weapon such as a gun, knife, or club on school property?			**2.2 (1.1–4.5)**
Yes	13%	3%	
No	87%	97%	

*Adjusted for gender, race/ethnicity, and grade level. Bolded values are statistically significant

In the multivariable logistic regression adjusted for gender, race/ethnicity and grade level, students who witnessed GV were 4.6 times more likely to report never feeling safe from violence in the community (95% CI 1.7–12.4). A third of the students who witnessed GV reported never feeling safe from sexual harassment in public spaces. The odds of a student never feeling safe from sexual harassment was 3.7 times higher for students who witnessed GV compared to those who had not (95% CI 1.5–8.8). Thirty-seven percent of the students who witnessed GV reported local transportation areas were never safe, compared to 22% of students who had not witnessed GV (OR 2.5, 95% CI 1.1–5.6). Not all questions regarding community safety differed based on exposure to GV: students reported similar rates of feeling that most of the time there are safe spaces to play and relax nearby and that most of the time the play spaces are in good condition. Almost a quarter of students who witnessed GV reported feeling they can never trust police (24%) compared to 14% of students who did not witness GV, but the difference was not statistically significant (OR 1.9, 95% CI 0.7–4.7).

There were differences in student perceptions of safety at school. Thirty percent of students who witnessed GV reported not attending school because the students felt they would be unsafe at school (OR 1.6, 95% CI 1.1–2.6). Only 19% of students who witnessed GV reported that they felt security guards at the school kept them safe most of the time, compared to more than 40% of the students who had not witnessed GV (OR 0.6, 95% CI 0.5–0.9). Students who witnessed GV had 2.2 times the odds of reporting carrying a weapon to school compared to those who had not witnessed GV (95% CI 1.1–4.5). Students who witnessed GV were less likely to report that there are adults at the school who they can talk to freely about abuse or violence most of the time (30% compared to 49%, OR 0.7, 95% CI 0.5–0.9).

## Discussion

The epidemiology of GV indicates that the USA has failed to grant its citizen^1^ children the basic human right to be protected from GV (Shah 2022). The study was undertaken with the ultimate goal of honoring children’s right to live free from violence and to have their views on the issues of safety and violence heard and respected. To achieve this goal, we centered the work within a Child Rights framework and engaged youth as co-researchers using a YPAR approach. Our study aims were to better understand youth perceptions of safety in EH and to explore relationships to GV exposure and demographic characteristics. The findings demonstrated that students were aware they have rights and highlighted a number of areas for possible interventions to improve the safety of children attending a school in EH and may have implications for similar urban environments.

### The pandemic and underserved communities

The context of this study is important. This research took place during the COVID-19 pandemic in EH, New York, a predominantly Latinx, underserved community. It has been well documented that low-resourced, minority communities have suffered more severe consequences of the pandemic both in terms of increased infections and deaths as well as an excess burden of violence (Gibbs et al. 2022; Do and Frank 2021). The increase in GV may be attributed to various pandemic related factors, including a rise in sales of handguns, pandemic related job losses, stagnant wages, rising inflation, increased stress and deteriorating mental health (Goldstick et al. 2022). These factors are reflected in our survey results: almost a third of students shared a parent or adult in the home who lost a job during the pandemic and a third of students reported having poor mental health most of the time or always.

### Gun violence epidemic impacts children’s perceptions of safety

There is an epidemic of firearm-related violence (Goldstick et al. 2022; Lee et al. 2022a). The USA has no peer country experiencing a similar crisis. Despite having similar rates of non-fatal crime and violence, the rate of firearm homicides in the USA is estimated to be 25 times higher than other high-income countries. (Shah 2022; Grinshteyn and Hemenway 2019). These data are not new—the public has been aware of the trend in GV for decades, yet comprehensive policies to combat the GV epidemic have not been implemented or enforced successfully. Everytown For Gun Safety, the largest GV prevention organization in America, has lauded New York as a national leader in GV prevention, because New York has passed some of the strongest gun laws in the country (Everytown 2022). Despite such achievements, we believe that this study demonstrates that more is needed in regard to GV prevention in New York including comprehensive approaches to tackling the continuing epidemic. It is unacceptable and counter to the framework of child rights that more than one third of the students surveyed in this study have personally witnessed GV and more than half have witnessed other forms of violence.

Our study highlights that witnessing GV was not detrimental to children’s understanding of their basic human rights nor their involvement in efforts to effect change within the community. However, witnessing GV was associated with feeling less safe in public spaces and at school. Survey responses suggested that students who witness GV were at a significant disadvantage compared to their peers: they were more likely to skip school because they felt unsafe, and to carry a weapon to school, and they were less likely to feel security guards kept them safe, and to feel that there were adults they could talk to about violence. Exposure to GV was also associated with a more negative perception of the community. Students who witnessed GV were more likely to report never feeling safe from sexual harassment, from air pollution, from violence in the community, or from dangerous situations in transportation waiting areas. Survey results also highlight there is a gender component that must not be ignored on issues of safety. Female students were significantly more likely to feel unsafe compared to their male peers: they were more likely to report feeling unsafe in transportation waiting areas and much more likely to experience sexual harassment.

### Implications: potential interventions to respect children’s rights to be protected from gun violence

1. Ratify the CRC

The CRC is the most widely ratified human rights treaty in history and is ratified by all members of the United Nations except the USA. In 1995, the US Ambassador to the United Nations signed the Convention, but no presidential administration since then has submitted the Convention to the Senate for ratification (Rutkow and Lozman 2006). If the USA were to ratify the CRC, not only would the USA strengthen credibility when advocating for children’s rights abroad*,* the USA would be required to confront hard truths about the areas in which children are denied basic human rights in the USA. Ratification of the CRC would put pressure on politicians to bring our laws and practices in line with human rights, with the support of the United Nations. In the meantime, and in support of the ultimate goal of implementing the CRC, initiatives such as UNICEF USA's Child Friendly Cities Initiative can help local governments put the child rights framework into action, in turn, building safe, equitable, just, inclusive, and child-responsive cities and communities.

2. Invest in effective implementation of gun laws

New York has already passed many of the most important policy strategies to combat GV, including requiring background checks on all gun sales, passing a strong Extreme Risk law, having strong permit systems for the purchase and carry of handguns, as well as enacting police reform (Everytown 2022). However, GV continues to be a major burden on the state and people of New York, and costs the state an estimated $11.4 billion every year (Everytown 2022). New Yorkers, especially the children of New York, would benefit from more comprehensive strategies to enforce the Gun Reform laws that have been passed and to collaborate across states to create multi-prong efforts to substantively address this epidemic. The American Academy of Pediatrics has laid out specific policy positions that could move closer to achieving this aim despite the lack of a nationally cohesive agenda to reduce this most lethal but preventable injury causation (Lee et al. 2022b). Other countries have taken additional measures to reduce the number and types of guns available to individuals (Tebor 2021; Anglemyer and Beautrais 2019).

3. Allocate Funds for improvements to public spaces, especially transportation waiting areas

A significant percentage of students surveyed in this study reported never feeling safe from sexual harassment and never feeling safe in transportation waiting areas. The NYC government has enacted a number of reforms to improve public transportation (Muennig et al. 2014). This survey reflects some success in that most students felt safe when riding on buses, however, too many students felt unsafe when waiting for transportation. After a mass shooting event occurred on a train in Brooklyn during April 2022, the week after this survey was administered, the NYC government responded by increasing police presence around transportation waiting areas (Ostapiuk 2023). Unfortunately, such actions may not make students feel safer in these public spaces, given that some of the vulnerable students surveyed reported never trusting police or feeling police protect them. Discussions with student interns involved in this research yielded the suggestion that a better use of public funds would be to improve the lighting and physical spaces of transportation areas. Improving community relations with police would also merit attention.

### Study limitations

This study was limited by a small sample size from a single high school. A larger sample size and oversampling for underrepresented groups would likely have allowed for analysis of survey responses without collapsing the data and analysis of possible interactions of variables such as gender and GV exposure and differences by self-identified racial/cultural descriptions therefore yielding a more nuanced and specific interpretation of the data (e.g. for our Asian American and Native/Alaskan populations). In addition, coupling the survey administration with focus group discussions would allow further illumination of youth concerns raised by the survey. Despite the small sample size, a strength of the study was the completion rate of the survey: students selected “prefer not to answer” only 3% of the time. The majority of skipped responses may have been due to survey fatigue from a small number of students since 9 students (6%) elected to skip more than 20% of the questions. The near-peer mentoring sessions, which were consistently attended, afforded the high school students direct mentorship from the graduate students, and while the high school students did attend sporadically the bi-weekly Zoom meetings with the larger advisory group, student schedules did not allow consistent participation. Our project had to abide by the principle of not interfering with the education of the students. Although the survey responses are from a single high school, and therefore not generalizable to all contexts, the results are still useful for comparison in urban environments similar to EH, and the methodology of employing YPAR within a Child Rights framework could be successfully employed with modifications to gather youth input from any location. We involved three students from the high school in the research process. Our intent was to have the students represent the school, but three students (two females, one male) cannot fully represent all youth voices from the high school or community. We worked with the three interns to try and make sure every question was worded appropriately, but there are limitations to the analysis based on the final wording of some questions. The lockdown imposed by the pandemic precluded a student suggestion in 2020–2021 to present the data to school assemblies for each grade. We will endeavor, however, to work with the school leadership to comply with educational law and respond to students’ desire to share their views more broadly. Additionally, the question “I have personally witnessed gun violence” is intentionally broad so as to give students the ability to determine for themselves if they feel they witnessed GV; however, the vagueness of the question can result in varied responses: one student might consider witnessing GV as hearing gunshots, while another may only consider personally witnessing GV if the student was physically in front of a person when the person was harmed by a firearm.

## Conclusions

The students in this study affirmed their rights as youth to participate and express their views on matters that may affect them, as articulated in the CRC. The study evinces the efficacy of employing a youth participatory action research methodology to identify and answer youth concerns of community safety and prioritizes honoring child rights in discerning what youth perceive to be critical to their well-being, in all contexts—physical, social, and emotional. The study identified gun violence among other exposures as a major contributor of East Harlem youth’s perception of lack of safety in their communities. The study revealed differences in perceptions of safety by self-identified gender. More research is needed to understand the possible differences by cultural/racial identity and experience of racially motivated violence. The findings compel choosing methods for youth to directly inform decision-makers of their views and compel the development of policies and interventions which consistently and uniformly require youth participation and direct input.

## Data Availability

The datasets used and/or analyzed during the current study are available from the corresponding author on reasonable request.

## Abbreviations

CDC: Centers for Disease Control and Prevention
CERG: Children’s Environmental Group
CFCI: Child Friendly Cities Initiative
CUNY: City University of New York
COL: Child Opportunity Level
CRC: Convention on the Rights of the Child
EH: East Harlem
GV: Gun violence
IAACT: Intergenerational Action Adolescent and Child Team
THS: The Heritage School
UN: United Nations
UNICEF: United Nations Children's Fund
USA: United States
YPAR: Youth Participatory Action Research
YRBSS: Youth Risk Behavior Surveillance Survey

## Footnotes

1 The word citizen is used to signify all children in the community regardless of documentation status.
